# RF Power, B_1+rms,_ and SAR Variation with RF Coils, Conductive Metallic Implants, and Ionic Solutions at 1.5T and 3T

**DOI:** 10.3390/s26185894

**Published:** 2026-09-17

**Authors:** David H. Gultekin

**Affiliations:** Rockefeller Neuroscience Institute, Department of Neuroradiology, School of Medicine, West Virginia University, Morgantown, WV 26506, USA; david.gultekin@aya.yale.edu

**Keywords:** RF power, B_1+rms_, SAR, RF coils, RF heating, temperature, metallic implants, ionic solutions

## Abstract

**Highlights:**

Access to magnetic resonance imaging (MRI) for patients with active and passive implants is restricted due to radiofrequency (RF) safety concerns. Ongoing research aims to evaluate the utility of RF power, time-averaged root-mean-square RF field (B_1+rms_), and specific absorption rate (SAR) for implant safety.

**What are the main findings?**

- MRI is remotely sensitive to metallic wavelengths and ionic concentrations.
- RF power is sensitive to the presence or absence of conductive metallic implants and ionic solutions, implant wavelengths, ionic concentrations, RF coils, and Tx/Rx combinations, whereas B_1+rms_ is not sensitive.

**What are the implications of the main findings?**

- RF power may be monitored because scanner-reported SAR may vary unpredictably with metallic and ionic conductivities.
- Saturation bands increase RF power, B_1+rms,_ and SAR, and they must be turned off when imaging patients with conductive metallic implants.

**Abstract:**

Currently, the time-averaged root-mean-square RF field (B_1+rms_) and specific absorption rate (SAR) are being evaluated to monitor and control RF-induced heating near conductive metallic implants, such as deep brain stimulation (DBS) leads, during MRI. However, experimental methods to assess the relationship between RF power, B_1+rms_, and SAR are lacking for RF coils, RF pulse sequences, metallic implants, and ionic solutions. A method is developed to evaluate the variation in RF power, B_1+rms_, and SAR with RF coils, metallic implants, and ionic solutions using phantoms consisting of water (H_2_O) and sodium chloride (NaCl) with four ionic concentrations (0, 1, 2, 3%), four metallic wavelengths (0, *λ*/2, *λ*, 2*λ*), two RF coils (body, head), transmit/receive (Tx/Rx) combinations, and five RF pulse flip angles (30, 45, 60, 75, 90 degrees) in two *B*_0_ fields (1.5T and 3T). The scanner-reported RF power and SAR varied with RF pulse sequences, RF coils, Tx/Rx, metallic implants, and ionic solutions, whereas B_1+rms_ varied only with RF pulse sequences. The RF power, B_1+rms_, and SAR relationship depends on RF pulse sequences, RF coils, Tx/Rx, metallic implant wavelengths, and ionic concentrations. SAR (whole-body, head) scaled with RF power by absorption ratios (*α*) variable with experimental conditions.

## 1. Introduction

There is a growing number of patients with conductive metallic implants and electronic devices that require magnetic resonance imaging (MRI) for diagnosis and therapy response monitoring. More than 4.6 million hip and knee procedures, including primary and revision arthroplasty procedures, were performed between 2012 and 2024 [1]. More than 400,000 patients in the US and about 1.7 million patients in the world receive cardiac implantable electronic devices (CIEDs) every year [2,3]. Up until now, it is estimated that more than 300,000 patients have received deep-brain stimulation (DBS) leads globally, and the number is increasing with time at a rate of about 12,000 per year [4]. The applications of electrical stimulation in neuroscience and neurosurgery have led to important advances in surgical treatments of neurological diseases and continue to grow in a wide range of neurological conditions [5,6,7,8,9,10,11,12,13].

However, conductive metallic implants may heat up significantly during the MRI and create safety risks for the patients [14,15,16,17,18]. The time-varying electromagnetic fields in the MRI may induce heating in the leads and potentially damage the cells in the vicinity of the leads implanted in the tissues [19,20,21,22,23]. Therefore, it is important to develop safety models and methods to predict and control the magnitude of heating in conductive implants during the MRI [24,25,26].

In general, most adverse effects reported about MRI involve thermal injury to the patients [27], through possible effects caused by electromagnetic field interactions [28,29], and catastrophic incidents in the presence of conductive metallic implant leads [30]. As a consequence, the potential applications of MRI are limited partly by considerations of radiofrequency (RF) safety that impose constraints on the design of RF coils and RF pulse sequences [31]. A current dilemma in MRI is how to optimize image quality and scan time while maintaining RF safety. Often, one factor may be improved at the expense of other factors.

There is a growing need and interest in assessing the utility of temperature rise (dT), specific absorption rate (SAR), and the time-averaged root-mean-square RF fields (B_1+rms_) to monitor RF absorption and thermal hazards in tissues, especially in the presence of conductive metallic implants such as DBS electrodes [32,33,34,35,36]. In a previous study, the relationship between RF-induced heating and temperature rise near a conductive metallic DBS lead, and the scanner-reported B_1+rms_ and RF power for RF pulse sequences, RF coils, and transmit and receive (Tx/Rx) combinations was investigated at 3T [37]. It was demonstrated that both temperature rise and RF power varied with RF pulse sequences, RF coils, and Tx/Rx combinations, whereas B_1+rms_ varied only with RF pulse sequences but not with RF coils and Tx/Rx combinations at 3T. The RF-induced heating and temperature rise were associated with RF power propagation and loss corresponding to the B_1+rms_ for RF coils and Tx/Rx combinations at 3T. The study also showed the RF heating effects of RF coils and Tx/Rx combinations with RF adjustments, including *B*_1_ shimming at 3T [37,38,39,40].

However, transient temperature measurements are invasive, complicated, and take a much longer time, as the temperature increases during the heating period over the scan duration and decreases during the cooling period. Experiments on in vivo and ex vivo brain tissue, with and without conductive implants, show that the magnitude of RF-induced heating and temperature rise increases with frequency, power, and duration, necessitating relatively long cooling times between experiments [20,41]. There is also global heating and melting of the gel medium in a finite-size phantom with repeated experiments, especially with higher frequency, power, and time, causing thermal convection and distortion of the temperature profile, which may require longer cooling time to return to baseline after several RF-intensive experiments [20,41]. During the MRI, temperature changes (increases when RF pulses are on and decreases when RF pulses are off), while RF power and B_1+rms_ stay constant.

In this study, the specific aim is to assess the relationship between the three scanner-reported noninvasive parameters, RF power, B_1+rms_, and SAR, varying with RF pulse sequences, RF coils, Tx/Rx combination, conductive metallic implant wavelengths, and ionic concentrations at 1.5T and 3T. This study also extends the previous study by comparing the effects of RF adjustments at 1.5T and 3T. This method can be used to compare experimental measurements with computational electromagnetic and thermal modeling to study RF coils, RF pulse sequences, and conductive metallic implants and media for RF-induced heating during MRI.

## 2. Materials and Methods

### 2.1. Theory

In nuclear magnetic resonance imaging, nuclear spins are placed in a static magnetic field (*B*_0_) and perturbed away from the equilibrium magnetization by an external transverse RF magnetic field (*B*_1_), and the resultant signal is received and processed spatially and temporally using a set of gradient coils and radiofrequency coils [42,43,44].

The relationship between the nuclear spin phase (*θ*) and the magnetic field (*B*_1_) can be derived from Larmor’s equation [42,43].

(1) $$\begin{array}{cc} \omega = & \gamma B_{1}\\ \theta = & \omega t_{p}=\gamma B_{1}t_{p} \end{array}$$

where *ω* is the angular Larmor frequency (rads/s), *γ* is the angular gyromagnetic ratio (rads/s·T), *B*_1_ is an external transverse magnetic field (Tesla), and *t_p_* is the pulse length (s). A plot of *B*_1_ vs. *θ* yields a straight line with a slope proportional to 1/*γt_p_*, which can be measured experimentally.

The specific absorption rate in an ex vivo tissue or a substance in the absence of perfusion can be related to the electric field or the initial rate of temperature rise at a point in the body, as

(2) $$SAR=\frac{\sigma E^{2}}{\rho }\;SAR=C_{p}{\left.\frac{\partial T}{\partial t}\right|}_{t\to 0}$$

where *σ* is the electrical conductivity (S/m), *E* is the root-mean-square (rms) electric field (V/m), *ρ* is the mass density (kg/m^3^), *C_p_* is the specific heat (J/kg·K) in tissue, and (∂*T*/∂*t*)_(*t*→0)_ is the initial temperature rise with time (K/s) [45].

The whole-body average SAR can be related to the average incident RF power (*P*) as

(3) $${SAR}_{wb}=\frac{P_{abs}}{m}\;P=\frac{m}{\alpha }{SAR}_{wb}$$

where *m* is the patient’s weight (kg) and *α* = *P_abs_*/*P* is a calculated dimensionless parameter called the absorption ratio, the ratio of the absorbed power (*P_abs_*) to the incident power (*P*). The expected value of *α* is a fraction (*α* < 1, *P_abs_* < *P*) that increases between 0 and 1 (0 < *α* < 1) with increasing frequency. A linear regression of *P* vs. SAR_wb_ yields a straight line with a slope proportional to *m*/*α*, which can be measured experimentally.

In the simplest coil element, the *E* field and the incident RF power (*P*) in a loop of wire with a radius (*r*) can be formulated using Equations (2) and (3) as

(4) $$E=\frac{1}{2}\omega rB_{1}\;P=\frac{\sigma m\omega^{2}r^{2}B_{1}^{2}}{4\alpha \rho }$$

where *ω* is the angular frequency, and *B*_1_ is the RF field [46,47,48,49]. A linear regression of *P* vs. the square of the *B*_1_ field yields a straight line with a slope proportional to $\sigma m\omega^{2}r^{2}/4\alpha \rho$, which can be measured experimentally.

Further, by substituting Equation (1) into Equation (4) and scaling it with two dimensionless parameters, number of slices (*N_S_*) and duty factor (*D*), the absorbed RF power and incident RF power can be related to the flip angle (*θ*) as

(5) $$P_{abs}=\frac{\sigma m\omega^{2}r^{2}N_{S}\theta^{2}}{4\rho \gamma^{2}t_{p}T_{R}}\;P=\frac{\sigma m\omega^{2}r^{2}N_{S}\theta^{2}}{4\alpha \rho \gamma^{2}t_{p}T_{R}}$$

where *α* = *P_abs_*/*P* and *D* = *t_p_*/*T_R_* is the ratio of pulse length (*t_p_*) to the repetition time (*T_R_*) [46,47,48,49]. These formulations are very simplified, and the exact relation for power deposition depends on the much more complicated electromagnetic fields in RF coils, implants, the direction of the *B*_1_ field, and the coordinate system in the tissue [28,50].

RF coils for MRI require a complicated electromagnetic design and optimization. However, the simplest element of a conductor loop and a capacitor (LC) still makes up the unit element of arrays with up to 128 LC loops [31]. The relationship between the RF power, magnetic field, and electric field in a coil depends on a specific coil design and a number of parameters [31,51,52]. However, the RF power and *B*_1_ field can be monitored for a coil or a combination of coils experimentally. Here, the variation in RF power, B_1+rms_, and SAR is evaluated in various experimental conditions by varying the metallic wavelengths and ionic concentrations using the commercially available RF coils and RF pulse sequences in two MR systems.

### 2.2. MRI Systems and RF Coils

Two MRI systems (Magnetom, 1.5T Aera and 3T Prisma, 64 MHz and 128 MHz, ^1^H NMR, proton nuclear magnetic resonance frequencies, both Syngo MR XA30A, Siemens Healthcare GmbH, Erlangen, Germany) were used in this study. In a previous study, five RF coil combinations, two Tx/Rx volume coils, a circularly polarized head coil (CP-H) and a body coil (BC), and three Rx-only phased-array surface coils, a 20-channel head and neck coil (20-H/N), a 32-channel head coil (32-H), and a 64-channel head and neck coil (64-H/N), were evaluated at 3T Prisma [37]. In this study, a widely used combination of RF coils, a BC Tx/Rx, and a 20-H/N Rx, in two Tx/Rx combinations, was used at both 1.5T and 3T to reduce the number of experiments. When the 20-H/N coil was turned off, the Tx/Rx was BC/BC, and when the 20-H/N coil was turned on, the Tx/Rx was BC/20-H/N. The RF polarization modes were circular polarization (CP) and elliptical polarization (EP). The 1.5T system had a no-tune BC Tx/Rx with 16 rungs, CP Tx, and 26.1 kW peak power, and the 3T system had a no-tune BC Tx/Rx with 32 rungs, multi-channel 2 (MC-2), CP and EP Tx, and 43.2 kW peak power (17.5 kW and 25.7 kW for channels 0 and 1). The BC specifications (maximum applied *B*_1+_ and B_1+rms_) were 27 μT and 6.2 μT for 1.5T, and 30 μT and 3.6 μT for 3T. The BC Tx field attenuation (3 dB and 10 dB) from the isocenter was 0.18 m and 0.38 m for 1.5T, and 0.15 m and 0.25 m for 3T.

### 2.3. Aqueous Solutions of NaCl-H_2_O

Aqueous solutions of sodium chloride (NaCl) and water (H_2_O) with four concentrations (C) of 0, 1, 2, and 3% NaCl by weight, corresponding to molar concentrations of 0, 171, 342, and 513 mM (millimole per liter), and specific gravities (SG) of 1.000, 1.005, 1.013, 1.020 kg/L (kilogram per liter), respectively, were used. These electrolytes with monovalent ions also correspond to 0, 171, 342, and 513 mEq/L (milliequivalent per liter) of Na^+^ (cations) and Cl^−^ (anions). NaCl has a molecular weight of 58.44 g/mol (22.99 g/mol Na + 35.45 g/mol Cl) and a maximum solubility of 26.5% by weight or 4.53 M (mol/L) in H_2_O. The electrical conductivity (σ) of electrolytes varies with temperature, ionic concentration, and frequency [53,54]. Additionally, conductivity artifacts, where the RF signal decays from the perimeter to the center of the phantom, increase with ionic concentration [54]. For reference, σ values for the deionized water with 0, 1, 2, and 3% NaCl-H_2_O solutions were measured as 0.0016, 18.4, 34.1, and 48.5 mS/cm (milli Siemens per centimeter), respectively, using a conductivity meter (InLab 731-ISM, Mettler-Toledo, Columbus, OH 43240, USA). Measurement of the conductivity of electrolytes in the RF range requires a specialized method and costly instrumentation not readily available [53]. Here, MRI is also being tested as a remote sensor for RF coil loadings with different ionic concentrations at the RF frequencies of 64 MHz and 128 MHz.

For comparison, normal saline is 0.9% NaCl (9 g/L, 154 mM, 154 mEq/L Na^+^ and Cl^−^) and is used for the treatment of dehydration and fluid imbalance, and the hypertonic saline is 3% NaCl (30 g/L, 513 mM, 513 mEq/L Na^+^ and Cl^−^), and is used for treatment of hyponatremic encephalopathy, traumatic brain injury, and cerebral edema. The human serum has a normal range of 135 to 145 mEq/L Na^+^.

### 2.4. Phantoms

A phantom consisting of a conductive metallic wire immersed in a conductive ionic solution was used in this study. Since the implant materials, designs, and positions affect RF-induced heating, the same implant with different wavelengths was used to reduce the number of experiments [55,56,57,58]. Any conductive metallic wire, such as a DBS lead or any other wire, such as a 14-gauge (Ga) wire, can be used in this method, although with a different relationship between RF power, B_1+rms,_ and SAR. A 14 Ga stranded wire with 64 strands, strand diameter of 0.2 ± 0.008 mm, copper-clad aluminum (CCA), 8% copper (Cu) cladding, 92% aluminum (Al) core, 4.1 μm Cu clad thickness, outside diameter (OD) of 1.85 mm, polyvinylchloride (PVC) insulation, average/minimum thickness of 0.425/0.375 mm, OD of 2.7 ± 0.1 mm, and an electrical resistance (R) of 14.1 Ohm/km) was used.

The penetration or skin depth (*δ*) of RF in a conductor is [59,60]

(6) $$\delta ={\left(\pi \sigma f\mu_{r}\mu_{0}\right)}^{-1/2}$$

where *σ* is the electrical conductivity (S/m), *f* is the RF frequency (Hz), *μ_r_* = *μ*/*μ*_0_ is the relative permeability of the material, the ratio of the permeability of the material (*μ*) to the permeability of free space (*μ*_0_).

The wavelength (*λ*) of RF in a medium is [61]

(7) $$\lambda =\lambda_{0}{\left(\varepsilon_{r}\right)}^{-1/2}$$

where *λ*_0_ = *c*/*f*_0_ is the wavelength in the vacuum, the ratio of the speed of light in vacuum (*c*) to the frequency in vacuum (*f*_0_), and *ε_r_* = *ε*/*ε*_0_ is the dielectric constant, the ratio of the permittivity of material (*ε*) to the permittivity of free space (*ε*_0_).

The electric permittivity and magnetic permeability of free space are related as

(8) $$\varepsilon_{0}={\left(\mu_{0}c^{2}\right)}^{-1}$$

where *ε*_0_ = 8.854 × 10^−12^ F/m (Farad per meter), *μ*_0_ = 4π × 10^−7^ H/m (Henry per meter), and *c* = 299,792,458 m/s (meter per second) as in Equation (8) [62].

Using the electrical conductivity (*σ*) of 5.96 × 10^7^ S/m for Cu and 3.77 × 10^7^ S/m for Al, and *μ_r_* of unity for Cu and Al in Equation (6), the RF skin depths at 64 MHz and 128 MHz were calculated as 8.15 μm and 5.76 μm for Cu and 10.3 μm and 7.25 μm for Al, respectively. The RF skin depth in both conductors at both frequencies is higher than the Cu clad thickness, making the conductivity of CCA wire a function of both conductors and both frequencies [63,64]. For reference, the conductivity of the CCA wire was calculated from its resistance as 3.5 × 10^7^ S/m.

The metallic wire wavelengths (*λ*) of 0*λ*, *λ*/2, λ and 2*λ* corresponding to lengths (L) of 0, 26, 52 and 104 cm (1.5T) and 0, 13, 26 and 52 cm (3T) in water (*ε_r_* = 81), as calculated by Equation (7), were used in combinations with the ionic concentrations (C) of 0, 1, 2 and 3% or 0, 171, 342, and 513 mM NaCl-H_2_O solutions.

**Figure 1:**
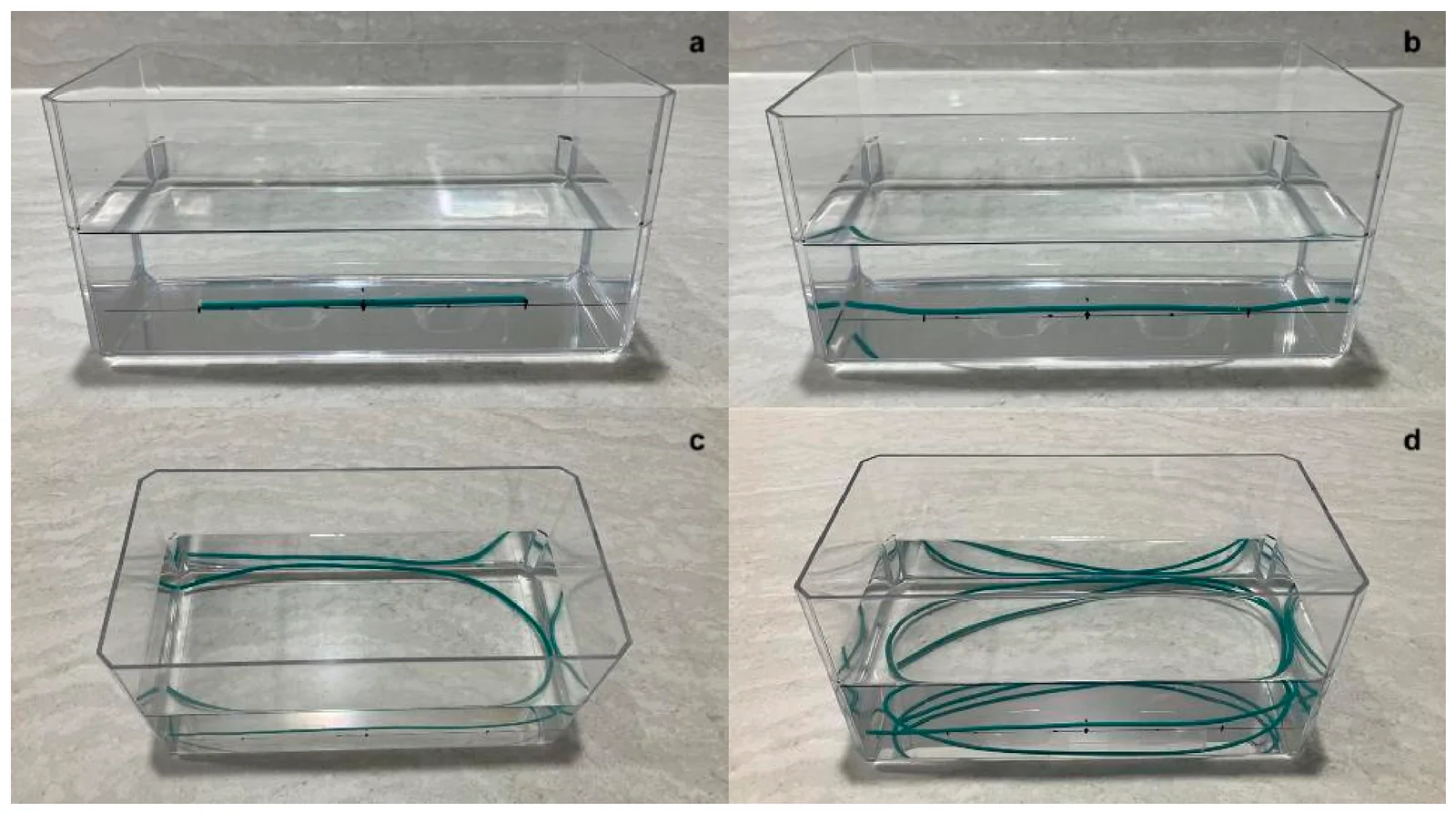
A phantom with conductive ionic solutions of NaCl-H_2_O, conductive metallic wire wavelengths of *λ*/2, *λ*, and 2*λ* corresponding to the metallic lengths of 13 cm (**a**), 26 cm (**b**), and 52 cm (**c**) at 3T or 26 cm (**b**), 52 cm (**c**), and 104 cm (**d**) at 1.5T.

As shown in Figure 1, the metallic wires of wavelengths of *λ*/2, *λ* and 2*λ*, corresponding to 13 cm (**a**), 26 cm (**b**), and 52 cm (**c**) at 3T or 26 cm (**b**), 52 cm (**c**) and 104 cm (**d**) at 1.5T, were attached at one end to the walls of poly methyl methacrylate (PMMA) plastic containers with dimensions of 22.86 cm, 11.125 cm, and 10.49 cm. A volume of 1.5L of each ionic solution with a height of 6.4 cm in the container was used, and the wire was positioned horizontally at 3.2 cm. The wire had a straight I-shape (**a**), a slightly curved C-shape (**b**), a U-shape (**c**), or a loop U-shape (**d**) in the phantom. The same metallic implant and trajectories were used in the phantoms when testing the ionic solutions, RF pulse sequences, and Tx/Rx combinations. The phantom with 0% NaCl and 0 *λ* or 0 cm implant had only deionized water and no wire.

### 2.5. RF Pulse Sequences

Five RF pulse sequences were prescribed to vary RF flip angles linearly as an independent parameter. A two-dimensional (2D) gradient echo (GRE) sequence with a variant of the fast low-angle shot (FLASH) sequence was modified with flip angles (*θ*) of 30, 45, 60, 75, and 90 degrees while keeping all other RF pulse parameters the same. The GRE sequences optimized with nominal parameters were taken from each of the scanners at 1.5T and 3T, respectively. The echo time (*T_E_*) was 4.76 ms and 2.46 ms, repetition time (*T_R_*) was 370 ms and 250 ms, slice thickness (*z*) was 5 mm and 4 mm, the total acquisition time (TA) was 69 and 77 s, pixel bandwidth (BW) was 189 Hz and 372 Hz at 1.5T and 3T, respectively. The number of slices (*N_S_*) was 3, the field of view (FOV) was 220 mm × 220 mm, and the matrix (m) size was 320 × 320. In the nominal sequences, an RF spatial saturation band (SB) of 50 mm with a 90-degree flip angle parallel to the axial slice was prescribed at 1.5T, but not at 3T. By default, the RF adjustment strategy was set to standard, *B*_0_ shimming was tune-up, *B*_1_ shimming was TrueForm, adjustment tolerance was auto, adjust with body coil was off, confirm frequency was never, and adjustment volume position was set to isocenter [65]. The RF adjustments, including *B*_1_ shimming (available at 3T but not at 1.5T), were performed by the scanner automatically [66]. Additionally, matched sequences with equal *N_S_*, SB, and *T_R_* were used to assess the difference between the nominal sequences at 1.5T and 3T. Since a previous study showed that RF power and the square of B_1+rms_ scaled linearly with three flip angles (30, 60, 90 degrees) and two RF pulse types (fast and normal), only one RF pulse type (fast) was prescribed in this study [37].

These five RF sequences were repeated for two RF coil Tx/Rx combinations (BC/BC and BC/20-H/N), four concentrations (C) of aqueous solutions of NaCl-H_2_O (0, 1, 2 and 3%) and four wavelengths (*λ*) of wires (0*λ*, *λ*/2, *λ* and 2*λ*) corresponding to four lengths (L) of wires (0, 26, 52 and 104 cm at 1.5T, and 0, 13, 26 and 52 cm at 3T).

The scanner-reported RF power, B_1+rms_, and SAR for the whole body and head (SAR_wb_ and SAR_h_) were recorded with high precision during the experiments. For consistency in SAR computation by the scanner’s algorithm, constant patient weight, height, and age (68 kg, 1.8 m, and 20 years) were used for all series. Additionally, transmit reference amplitude (TxRA) and transmit frequency (TxF) were recorded, and the absorption ratio (α) was computed from RF power and SAR_wb_ as *α* = *m*·SAR_wb_/*P* for each of the experiments.

### 2.6. Experimental Design and Statistical Analysis

A full factorial design of experiments (DOE) with multiple factors and levels was used to study the variation of the dependent variables (*P*, B_1+rms_, SAR, TxRA, TxF, and *α*) with independent variables (*θ*, Tx/Rx, L, and C). The experiments consisted of 5 levels of *θ* (30, 45, 60, 75, and 90 degrees), 2 levels of Tx/Rx combinations (1 and 2), 4 levels of L (0*λ*, *λ*/2, *λ* and 2*λ*), and 4 levels of C (0, 1, 2 and 3%), and a total of 160 experiments (5 × 2 × 4 × 4) per *B*_0_ field and 320 experiments (160 × 2) for both *B*_0_ fields (1.5T and 3T), with each session spanning over several hours. Multiple replicates were not performed due to the high number of experiments. The linear regression analyses were performed for B_1+rms_ vs. *θ*, *P* vs. the square of B_1+rms_, and *P* vs. SAR_wb_, for Tx/Rx, L, and C, respectively.

A fractional factorial DOE was performed to assess the variation in RF power, B_1+rms_, and SAR with the number of slices, saturation bands, Tx/Rx, and repetition time using a standard phantom with an aqueous solution of 5 g/L NaCl and 3.75 g/L NiSO_4_·6H_2_O (Siemens, 5300 mL) without an implant at both 1.5T and 3T. Additionally, experiments were performed with the same standard phantom to assess the effects of saturation band thickness and slice thickness on *P*, B_1+rms,_ and SAR using the same RF pulse sequences with three flip angles (30, 60, 90 degrees) and the Tx/Rx combination of BC/20-H/N.

## 3. Results

The scanner-reported time-averaged RF power (*P*), time-averaged root-mean-square RF field (B_1+rms_), and specific absorption rate (SAR) for the whole body (SAR_wb_) and head (SAR_h_) were evaluated as functions of flip angle (*θ*), RF coil Tx/Rx combinations (BC/BC and BC/20-H/N), metallic implant lengths (L), and ionic concentrations (C) of NaCl-H_2_O solutions. All linear regression and statistical analyses were based on the scanner-reported parameters.

The linear regression analyses were performed for B_1+rms_ vs. *θ*, *P* vs. the square of B_1+rms_, and *P* vs. SAR_wb_ for 5 RF pulse sequences. The linear regression parameters (slope and intercept) and the coefficient of determination (R^2^) were computed for 160 experiments, comprising 32 linear fittings each at 1.5T and 3T, respectively. The slopes of *dB*_1_/*dθ*, *dP*/*dB*_1_^2^, *dP*/*dS_wb_*, and the intercepts of *B*_1_(0), *P*(0), and *P*(0), along with the regression standard errors, are given in Table 1 and Table 2 for 1.5 and 3T, respectively. Here, RF power, B_1+rms_, SAR_wb_, and SAR_h_ are denoted by *P*, *B*_1_, *S_wb_*, and *S_h_* in the regression analysis.

The results (slopes, intercepts, and standard errors) for Tx/Rx combinations were identical in Table 1 but not in Table 2 due to the absence and presence of *B*_1_ shimming at 1.5T and 3T, respectively.

B_1+rms_ varied linearly with *θ*, proportional to Equation (1), whereas *P* and SAR varied linearly with the square of B_1+rms_ and the square of *θ*, proportional to Equations (4) and (5). The *dB*_1_/*dθ* did not change with any of the experiments as a function of Tx/Rx, L, and C, while *dP*/*dB*_1_^2^ and *dP*/*dS_w_* changed with experiments as a function of Tx/Rx (3T), L, and C, as given in Table 1 and Table 2. The difference in *dB*_1_/*dθ*, 3.5 nT/deg vs. 9.5 nT/deg in Table 1 and Table 2 was due to the differences in the nominal RF pulse sequences at 1.5T (50 mm SB and *T_R_* of 370 ms) and at 3T (no SB and *T_R_* of 250 ms), respectively. In Table 1, the intercept of *B*_1_(0) = 839 nT for water with no implant, corresponding to a flip angle of zero degrees, is the B_1+rms_ used for the SB in the nominal sequence at 1.5T for all the experiments.

The R^2^ values of linear regression of B_1+rms_ vs. *θ* were less than 1 (R^2^ < 1) in Table 1 because of the prescription of SB at 1.5T. The R^2^ values of linear regression were 1 for all fittings in Table 2 because of the absence of SB at 3T, except for one: the regression of B_1+rms_ vs. *θ* (L = 26 cm, C = 3% and Tx/Rx = BC/20-H/N). The B_1+rms_ for *θ* = 90° was less than usual (0.7855 μT vs. 0.8525 μT), and a regression of B_1+rms_ of 0.7855 μT vs. *θ* for this experiment calculated a *θ* = 83° instead of 90°, which can be confirmed by the linearity in the plot of *P* vs. *B*_1_^2^ for this experiment at 3T (red closed markers). The R^2^ values of linear regressions of *P* vs. *B*_1_^2^ and *P* vs. SAR_wb_ in Table 1 and Table 2 were all equal to 1 (R^2^ = 1) at 1.5T and 3T, respectively.

Table 3 shows the slopes and intercepts of *dB*_1_/*dθ* and *B*_1_(0) for *B*_1_ vs. *θ*, *dP*/*dB*_1_^2^ and *P*(0) for *P* vs. *B*_1_^2^, and the R^2^ for the experiments with matched RF sequences (*N_S_*, SB, Tx/Rx, and *T_R_*) at 1.5T and 3T, respectively. The slopes and intercepts, along with the R^2^, were computed by fitting *B*_1_ vs. *θ* and *P* vs. *B*_1_^2^ by varying *N_S_*, SB, Tx/Rx, and *T_R_*. The *N_S_*, SB, and *T_R_* prescribed in the RF pulse sequences had effects on the B_1+rms_, *P*, and SAR at both 1.5T and 3T.

As shown in Table 3, the B_1+rms_ varied linearly with *θ* proportional to Equation (1), independent of the magnetic field strength (*B*_0_), when the same pulse sequence parameters were used. The *dB*_1_/*dθ* was equal at 1.5T and 3T, independent of the *B*_0_ field, when SB was off, but not equal when SB was on. The variation in B_1+rms_ vs. *θ* was linear (R^2^ = 1) when SB was off, but nonlinear (R^2^ < 1) or quadratic (R^2^ = 1) when SB was on. The slope *dB*_1_/*dθ* and the intercept *B*_1_(0) varied distinctly when SB was on or off. The intercept corresponding to B_1+rms_ for zero-degree flip angle was zero, *B*_1_(0) = 0, when SB was off and nonzero, *B*_1_(0) ≠ 0, when SB was on. The slope, *dB*_1_/*dθ*, and intercept, *B*_1_(0), had a linear relationship when SB was on. The slope, *dB*_1_/*dθ*, decreased, and the intercept, *B*_1_(0), increased when SB was on.

RF power varied linearly with the square of B_1+rms_ and the square of *θ*, consistent with Equations (4) and (5), but with different slopes for each of the experiments as a function of Tx/Rx (3T), metallic wavelengths, and ionic concentrations, as in Table 1 and Table 2.

**Figure 2:**
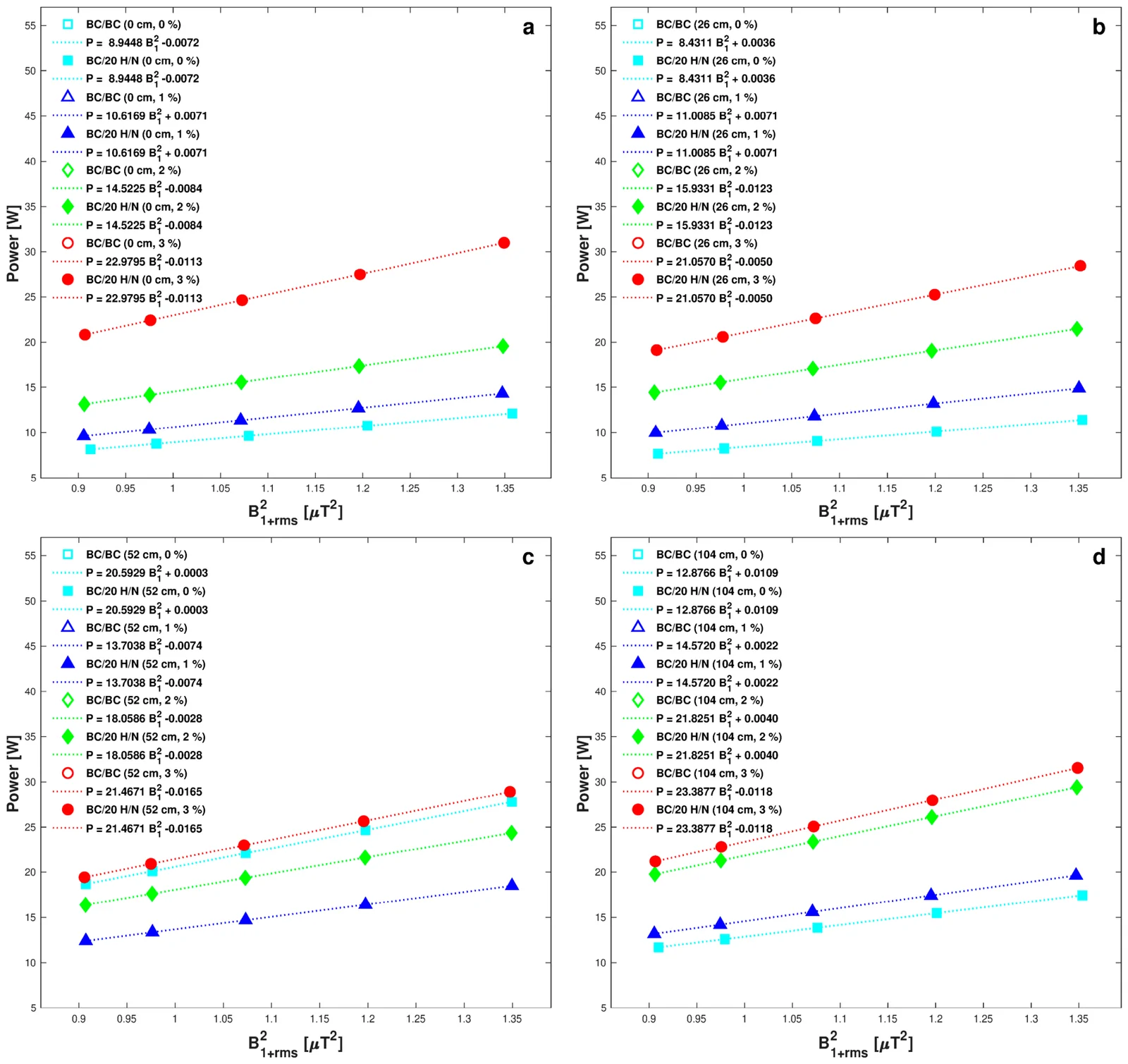
RF power vs. square of B_1+rms_ for NaCl-H_2_O solutions with four conductive ionic concentrations, 0% (**☐**), 1% (**Δ**), 2% (**♢**) and 3% (**〇**), four conductive metallic implant wavelengths, 0*λ* (**a**), *λ*/2 (**b**), *λ* (**c**), and 2*λ* (**d**), corresponding to four lengths, 0 cm (**a**), 26 cm (**b**), 52 cm (**c**) and 104 cm (**d**), and two Tx/Rx combinations, BC/BC (open markers) and BC/20-H/N (closed markers) at 1.5T. Note that closed markers are overlaid on open markers at 1.5T.

**Figure 3:**
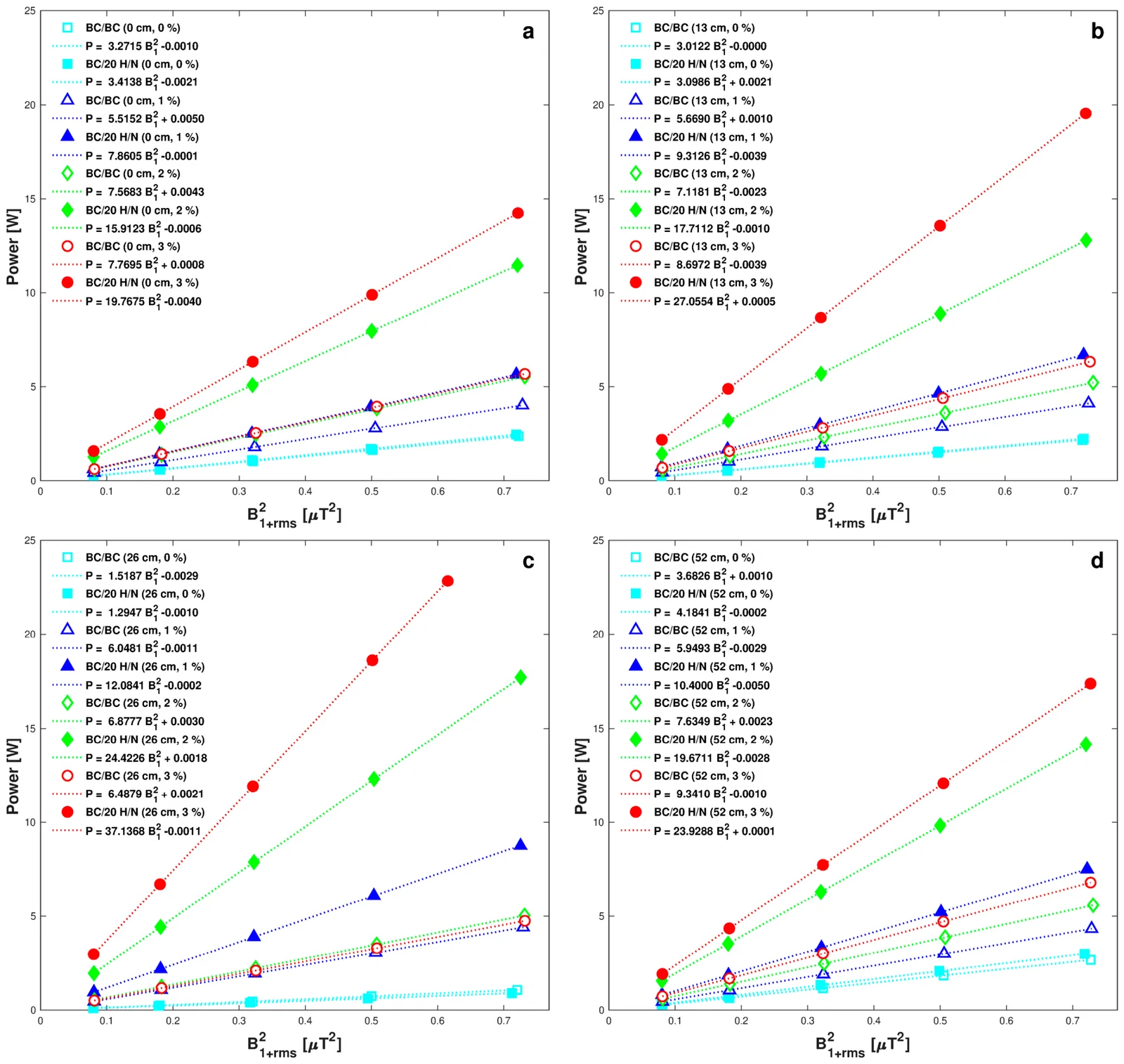
RF power vs. square of B_1+rms_ for NaCl-H_2_O solutions with four conductive ionic concentrations, 0% (**☐**), 1% (**Δ**), 2% (**♢**) and 3% (**〇**), four conductive metallic implant wavelengths, 0*λ* (**a**), *λ*/2 (**b**), *λ* (**c**), and 2*λ* (**d**), corresponding to four lengths, 0 cm (**a**), 13 cm (**b**), 26 cm (**c**), and 52 cm (**d**), and two Tx/Rx combinations, BC/BC (open markers) and BC/20-H/N (closed markers) at 3T. Note that closed markers are not overlaid on open markers at 3T.

The plots of *P* vs. the square of B_1+rms_ for NaCl-H_2_O solutions with NaCl concentrations of 0% (cyan squares, **☐**), 1% (blue triangles, **Δ**), 2% (green diamonds, **♢**) and 3% (red circles, **〇**) with metallic implant wavelengths of 0 (**a**), *λ*/2 (**b**), *λ* (**c**) and 2*λ* (**d**) corresponding to the lengths (L) of 0 cm (**a**), 26 cm (**b**), 52 cm (**c**) and 104 cm (**d**) at 1.5T, and 0 cm (**a**), 13 cm (**b**), 26 cm (**c**) and 52 cm (**d**) at 3T for Tx/Rx combinations of BC/BC (open markers) and BC/20-H/N (closed markers) with the linear regression lines (dotted lines) and linear regression coefficients (legends) are given in Figure 2 and Figure 3, respectively.

RF power varied linearly with whole-body SAR proportional to Equation (3) but with different slopes (*m*/*α*) and absorption ratios (*α*) for each of the experiments as a function of Tx/Rx (3T), metallic wavelengths, and ionic concentrations.

**Figure 4:**
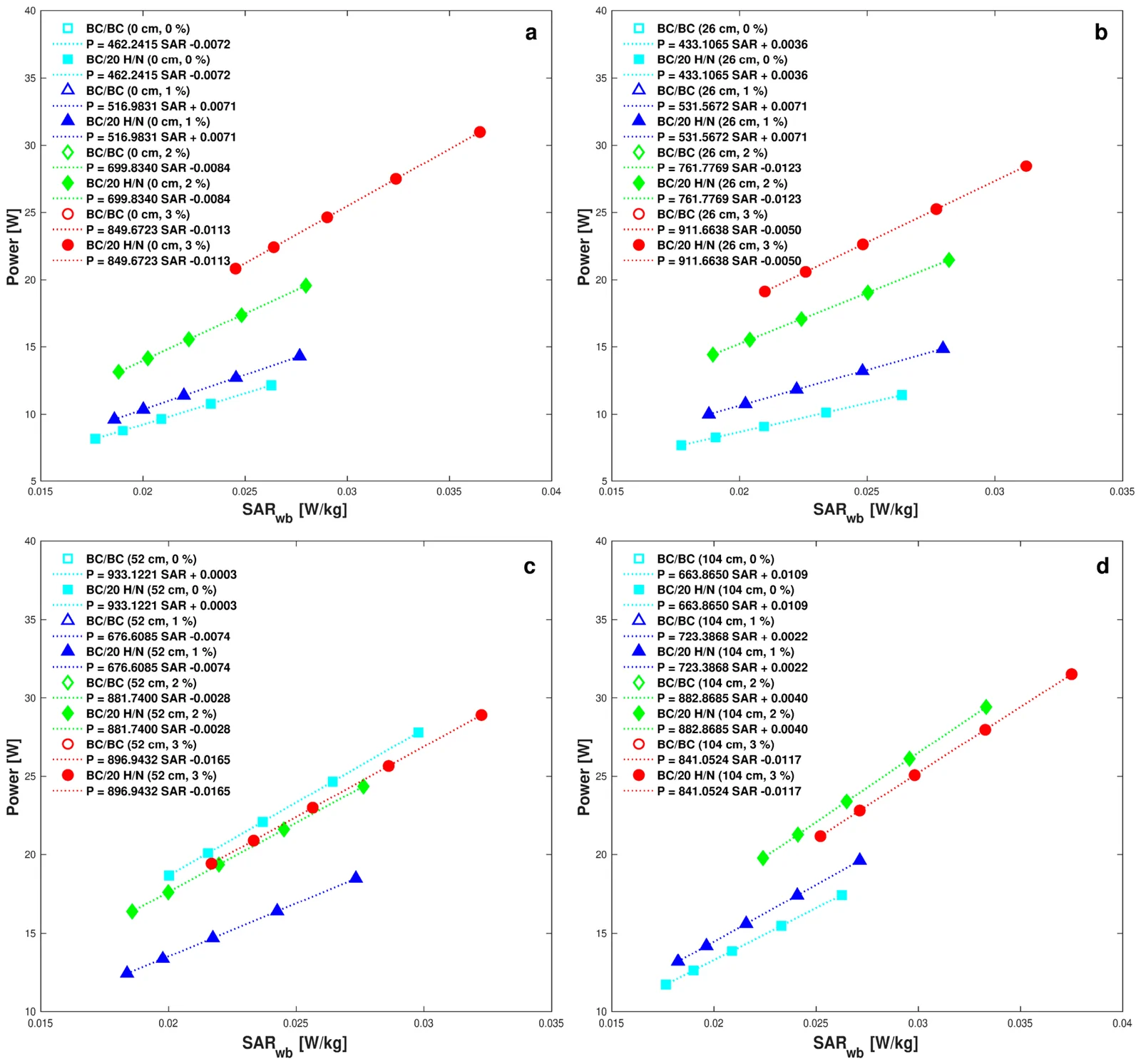
RF power vs. whole-body SAR for NaCl-H_2_O solutions with four conductive ionic concentrations, 0% (**☐**), 1% (**Δ**), 2% (**♢**) and 3% (**〇**), four conductive metallic implant wavelengths, 0*λ* (**a**), *λ*/2 (**b**), *λ* (**c**), and 2*λ* (**d**), corresponding to four lengths, 0 cm (**a**), 26 cm (**b**), 52 cm (**c**) and 104 cm (**d**), and two Tx/Rx combinations, BC/BC (open markers) and BC/20-H/N (closed markers) at 1.5T. Note that closed markers are overlaid on open markers at 1.5T.

**Figure 5:**
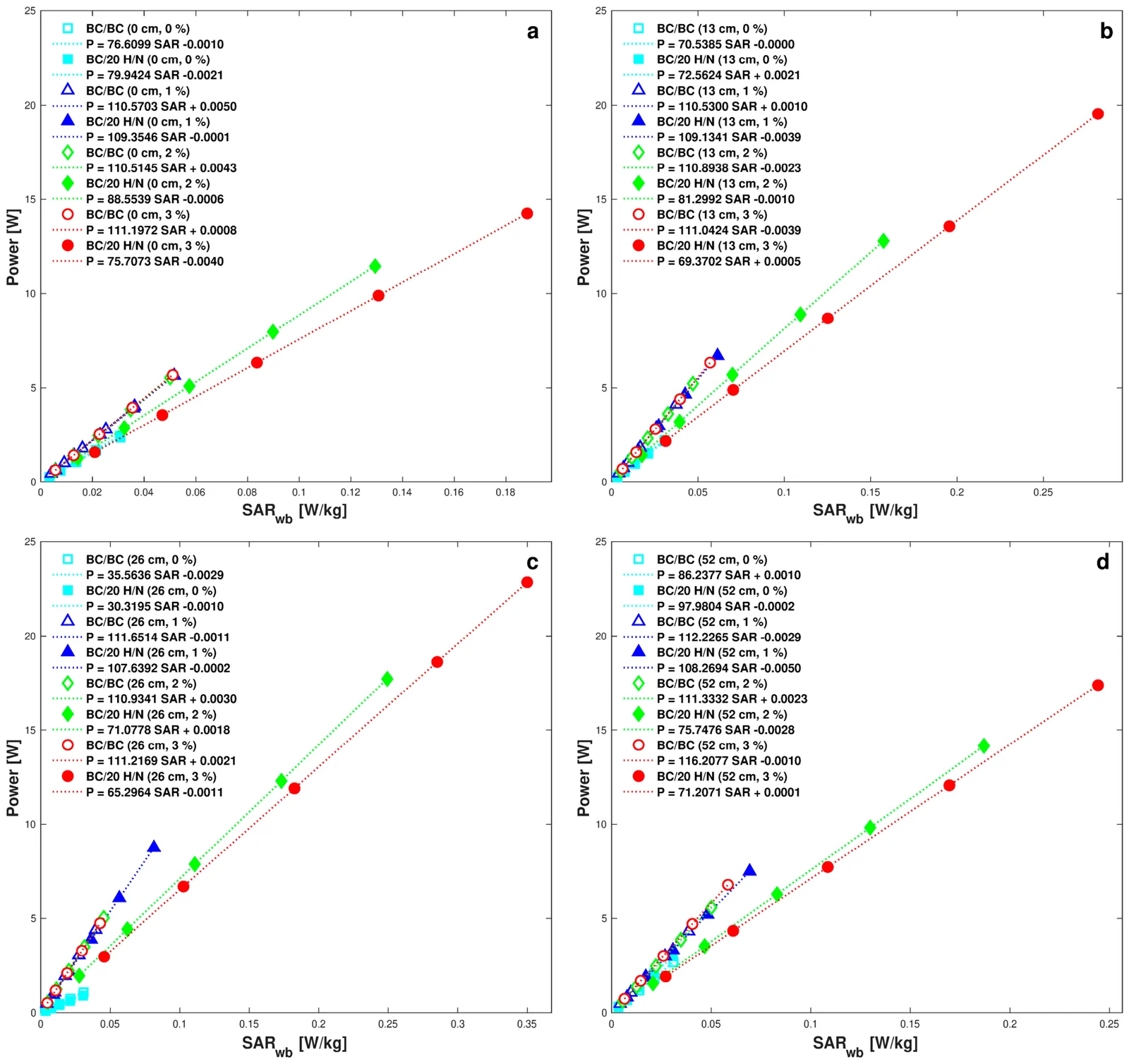
RF power vs. whole-body SAR for NaCl-H_2_O solutions with four conductive ionic concentrations, 0% (**☐**), 1% (**Δ**), 2% (**♢**) and 3% (**〇**), four conductive metallic implant wavelengths, 0*λ* (**a**), *λ*/2 (**b**), *λ* (**c**), and 2*λ* (**d**), corresponding to four lengths, 0 cm (**a**), 13 cm (**b**), 26 cm (**c**), and 52 cm (**d**), and two Tx/Rx combinations, BC/BC (open markers) and BC/20-H/N (closed markers) at 3T. Note that closed markers are not overlaid on open markers at 3T.

The plots of *P* vs. the SAR_wb_ for NaCl-H_2_O solutions with NaCl concentrations of 0% (cyan squares, **☐**), 1% (blue triangles, **Δ**), 2% (green diamonds, **♢**) and 3% (red circles, **〇**) with metallic implant wavelengths of 0*λ* (**a**), *λ*/2 (**b**), *λ* (**c**) and 2*λ* (**d**) corresponding to the lengths (L) of 0 cm (**a**), 26 cm (**b**), 52 cm (**c**) and 104 cm (**d**) at 1.5T, and 0 cm (**a**), 13 cm (**b**), 26 cm (**c**) and 52 cm (**d**) at 3T for Tx/Rx combinations of BC/BC (open markers) and BC/20-H/N (closed markers) with the linear regression lines (dotted lines) and linear regression coefficients (legends) are given in Figure 4 and Figure 5, respectively.

In Figure 2, Figure 3, Figure 4 and Figure 5, the plots with no metallic implants (L = 0 cm, upper left plots, **a**) show the effects of the conductive ionic concentrations (0, 1, 2, and 3% NaCl-H_2_O) and the plots with no ionic solutions (0% NaCl-H_2_O, cyan lines and square markers **☐**) show the effects of the conductive metallic implant wavelengths of 0*λ* (**a**), *λ*/2 (**b**), *λ* (**c**) and 2*λ* (**d**) (corresponding to lengths of 0 cm (**a**), 26 cm (**b**), 52 cm (**c**), and 104 cm (**d**) at 1.5T and 0 cm (**a**), 13 cm (**b**), 26 cm (**c**), and 52 cm (**d**) at 3T) on the RF power, B_1+rms_ and SAR_wb_ for RF coil Tx/Rx combinations of BC/BC (open markers) and BC/20-H/N (closed markers), at 1.5T and 3T, respectively.

Note that in Figure 2 and Figure 4, corresponding to 1.5T, there were no differences in Tx/Rx combinations (closed markers were overlaid on the open markers), whereas in Figure 3 and Figure 5, corresponding to 3T, there were differences in Tx/Rx combinations (closed markers were not overlaid on the open markers) in plots of *P* vs. *B*_1_^2^ and *P* vs. SAR_wb_, respectively. This difference is due to the absence and presence of B_1_ shimming at 1.5T and 3T, respectively.

The slopes of *dB*_1_/*dθ* varied slightly from 3.49 to 3.51 nT/deg at 1.5T and from 9.38 to 9.52 nT/deg at 3T across all experiments with varying metallic wavelengths and ionic concentrations (Table 1 and Table 2).

The slopes of *dP*/*dB*_1_^2^ varied from 8.4311 W/μT^2^ (Tx/Rx = 1 and 2, *λ*/2 = 26 cm, C = 0%) to 23.3877 W/μT^2^ (Tx/Rx = 1 and 2, 2*λ* = 104 cm, C = 3%) at 1.5T and from 1.2947 W/μT^2^ (Tx/Rx = 2, *λ* = 26 cm, C = 0%) to 37.1368 W/μT^2^ (Tx/Rx = 2, *λ* = 26 cm, C = 3%) at 3T with Tx/Rx, metallic wavelengths and ionic concentrations for all experiments (Table 1 and Table 2, and Figure 2 and Figure 3).

The slopes of *dP*/*dS_wb_*, equal to the ratio of patient weight to absorption ratio (m/α) proportional to Equation (3), varied from 433.1065 kg/α (Tx/Rx = 1 and 2, *λ*/2 = 26 cm, C = 0%) to 933.1221 kg/*α* (Tx/Rx = 1 and 2, *λ* = 52 cm, C = 0%) corresponding to *α* = 0.157 to *α* = 0.073 at 1.5T and from 30.3195 kg/*α* (Tx/Rx = 2, *λ* = 26 cm, C = 0%) to 116.2077 kg/*α* (Tx/Rx = 1, 2*λ* = 52 cm, C = 3%) corresponding to *α* = 2.243 to *α* = 0.585 at 3T, varying with Tx/Rx, metallic wavelengths, and ionic concentrations for all experiments (Table 1 and Table 2, and Figure 4 and Figure 5). The larger *α* values close to unity (*α* ≤ 1) indicate overestimation, and the outliers larger than unity (*α* > 1) indicate artifacts in overestimation of the whole-body SAR vs. average RF power at 3T, varying with RF adjustments, metallic wavelengths, and ionic concentrations.

The slopes of linear regressions of *P* vs. SAR for head and whole-body, *dP*/*dS_h_* and *dP*/*S_wb_* scaled with each other linearly, for the two Tx/Rx combinations of BC/BC and BC/20-H/N, with ratios of 0.1257 and 0.1257 at 1.5T and 0.1798 and 0.2223 at 3T, which were simply equal to the reciprocals of the ratios of SAR_h_ and SAR_wb_, with 7.9554 and 7.9554 at 1.5T and 5.5605 and 4.4977 at 3T, respectively, for all experiments varying with metallic implant wavelengths and ionic concentrations. There was no difference in Tx/Rx at 1.5T, but there was a difference at 3T in the SAR ratio. This was due to the differences in RF adjustments and *B*_1_ shimming at 1.5T and 3T, respectively.

Using the descriptive statistics, the mean plus and minus one standard deviation (m ± sd) calculated for all 160 experiments at each *B*_0_ field without replicates was 1.0467 ± 0.0750 μT and 0.5669 ± 0.2007 μT (B_1+rms_), 17.8827 ± 6.1112 W and 3.7476 ± 4.2007 W (RF power), 0.0241 ± 0.0045 W/kg and 0.0451 ± 0.0596 W/kg (SAR_wb_), 421.6163 ± 66.6073 V and 360.4076 ± 132.7007 V (TxRA), 0.0991 ± 0.0265 and 0.8270 ± 0.3613 (*α*) at 1.5T and 3T, respectively. Further tests with multiple replications may be necessary across MRI systems, RF coils, and implants.

The variation in dependent variables (RF power, B_1+rms_, whole-body specific absorption rate, transmit reference amplitude, transmit frequency, and absorption ratio) was evaluated as a function of independent variables (flip angle, RF coil transmit and receive combinations, metallic wavelengths, and ionic concentrations) for 1.5T and 3T.

**Figure 6:**
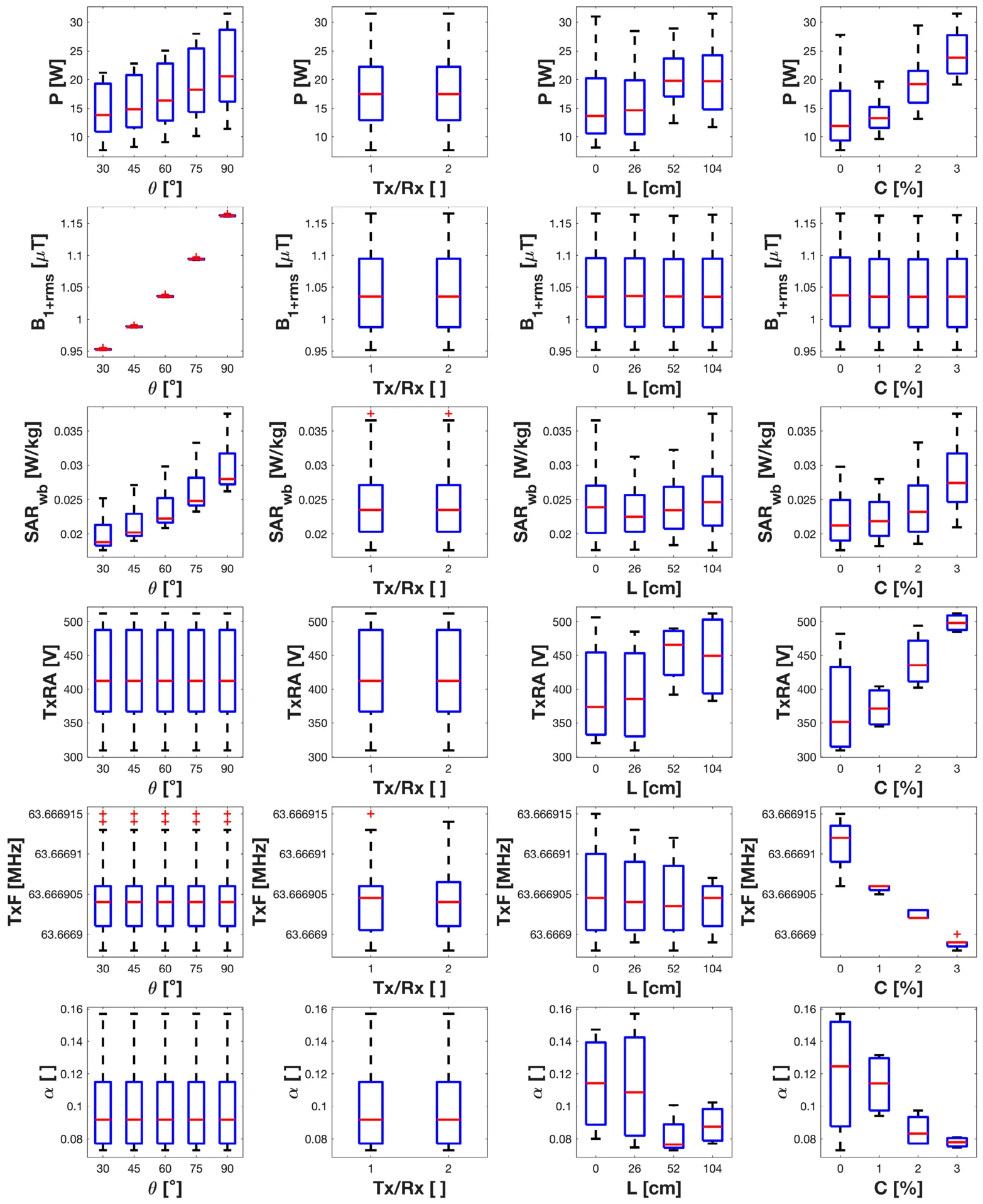
Boxplots of dependent variables of *P* (W), B_1+rms_ (μT), SAR_wb_ (W/kg), TxRA (V), TxF (MHz) and *α* (rows, from top to bottom, respectively) vs. independent variables of *θ* (30, 45, 60, 75, 90°), Tx/Rx (1: BC/BC, 2: BC/20-H/N), L (0, 26, 52, 104 cm) and C (0, 1, 2, 3%) (columns, from left to right, respectively) with statistics (minimum, first quartile, median, third quartile, maximum, and outliers) at 1.5T.

**Figure 7:**
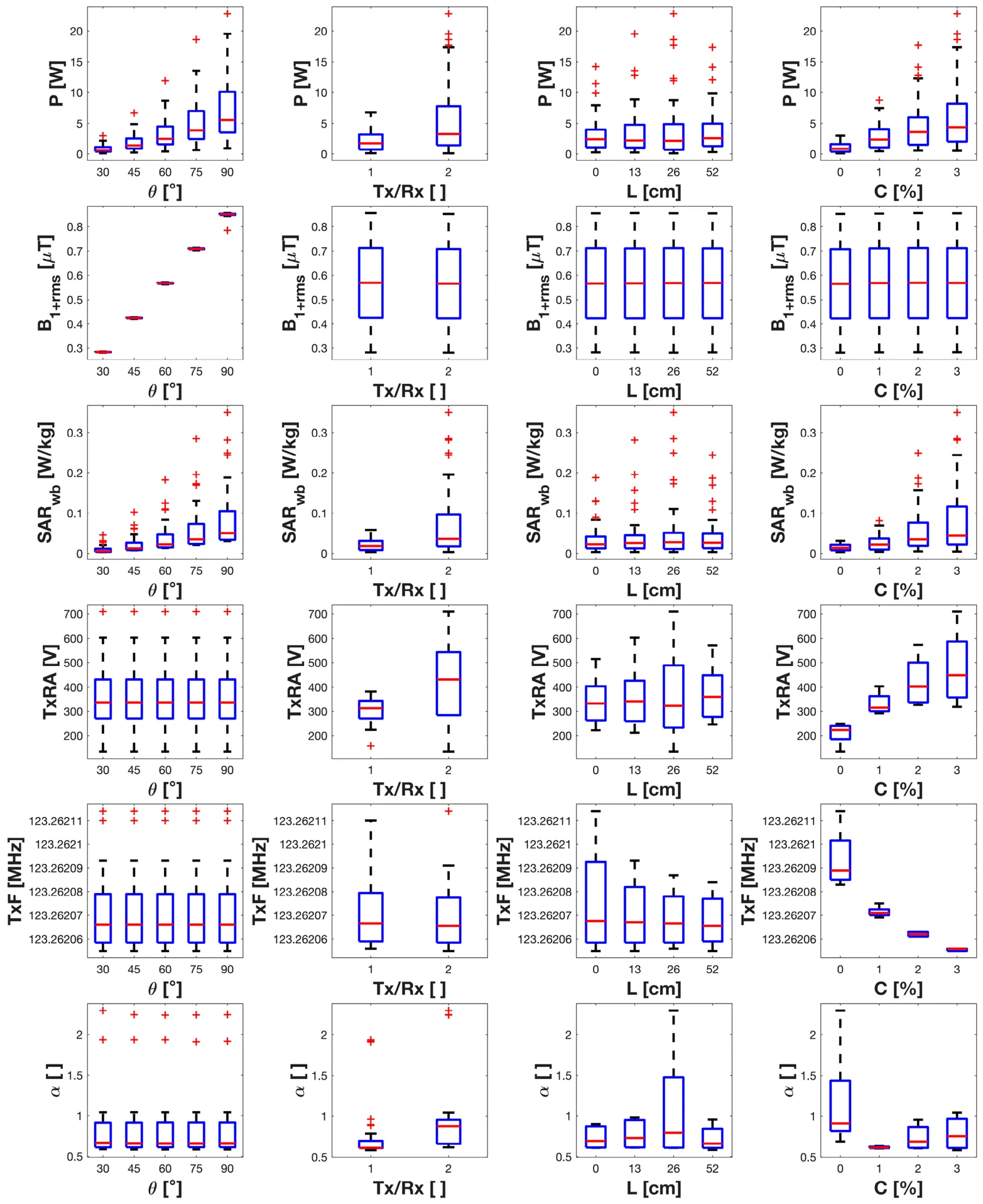
Boxplots of dependent variables of *P* (W), B_1+rms_ (μT), SAR_wb_ (W/kg), TxRA (V), TxF (MHz) and *α* (rows, from top to bottom, respectively) vs. independent variables of *θ* (30, 45, 60, 75, 90°), Tx/Rx (1: BC/BC, 2: BC/20-H/N), L (0, 13, 26, 52 cm) and C (0, 1, 2, 3%) (columns, from left to right, respectively) with statistics (minimum, first quartile, median, third quartile, maximum, and outliers) at 3T.

The boxplots of dependent variables of *P* (W), B_1+rms_ (μT), SAR_wb_ (W/kg), TxRA (V), TxF (MHz), and *α* (rows, from top to bottom, respectively) vs. independent variables of *θ* (30, 45, 60, 75, 90 degrees), Tx/Rx (1 and 2), L (0, 26, 52, and 104 cm at 1.5T, and 0, 13, 26, and 52 cm at 3T), and C (0, 1, 2, and 3%) (columns, from left to right, respectively) with statistics (minimum, first quartile, median, third quartile, maximum, and outliers) are given in Figure 6 and Figure 7 for 1.5T and 3T, respectively.

*P* and *B*_1_ both increased with SB while maintaining the *P*/*B*_1_^2^ nearly constant in a standard phantom without an implant, as in Table 3. The ratios of *P* and B_1+rms_ with and without SB, *P*_(SB)_/*P* and *B*_1(SB)_/*B*_1_, for five RF pulse flip angles (30, 45, 60, 75, 90 degrees) and equal sequence parameters (*N_S_* = 3, *T_R_* = 250 ms, Tx/Rx = 2) were 16.47, 7.84, 4.85, 3.47, 2.71 and 4.05, 2.80, 2.20, 1.86, 1.65 at 1.5T, and 11.31, 5.58, 3.56, 2.64, 2.31, and 3.36, 2.36, 1.89, 1.63, 1.46 at 3T, respectively. Note that the ratio of *P*_(SB)_/*P* scales with the square of the ratio of *B*_1(SB)_/*B*_1_ at both 1.5T and 3T.

*P* and *B*_1_^2^ varied linearly with *N_S_* and 1/*T_R_* as in Equation (5). Both *P* and *B*_1_ increased with *N_S_* and decreased with *T_R_* while maintaining a close ratio of *P*/*B*_1_^2^ in a standard phantom without an implant when turning the SB on or off at both 1.5T and 3T, as in Table 3. For example, the *dP*/*dB*_1_^2^ values for five RF pulse flip angles and equal sequence parameters (*N_S_* = 1, *T_R_* = 250 ms, Tx/Rx = 2) with and without SB (1st and 5th rows) were 9.24 and 9.24 W/μT^2^ at 1.5T, and 7.77 and 7.82 W/μT^2^ at 3T, respectively.

B_1+rms_ vs. *θ* varied with SB thickness in a step function where *dB*_1_/*dθ* took one value, 9.6 nT/deg, for an SB thickness of 0 mm, and one value, 6.6 nT/deg, for SB thicknesses of 3, 4, and 6 mm, and then another value, 4.9 nT/deg, for SB thicknesses of 12, 25, and 50 mm. The slice thickness (*z*), varied from 4 mm to 16 mm (4, 8, and 16 mm), did not have any effect on *P*, B_1+rms_, and SAR, and the linear regression coefficients, *dB*_1_/*dθ*, *dP*/*dB*_1_^2^, and *dP*/*dS_wb_*, for three flip angles (30, 60, and 90 degrees) at 1.5T and 3T.

*P* and B_1+rms_ increased with the thickness of SB in a step function while maintaining a close ratio of *P*/*B*_1_^2^ at 1.5T and 3T in a standard phantom without an implant. The ratios of power and B_1+rms_ with and without SB, *P*_(SB)_/*P* and *B*_1(SB)_/*B*_1_, for three flip angles (30, 60, 90 degrees) were 5.02, 2.00, 1.44, and 2.23, 1.41, 1.20 for SB thicknesses of 3, 4, and 6 mm, and 11.32, 3.57, 2.14, and 3.36, 1.89, 1.46 for SB thicknesses of 12, 25, and 50 mm, respectively. The *dP*/*dB*_1_^2^ were 8.06, 8.04, and 8.06 W/μT^2^ for SB thicknesses of 0 mm, and SB thicknesses of 3, 4, and 6 mm, and SB thicknesses of 12, 25, and 50 mm, respectively.

## 4. Discussion

In a previous study, the relationship between RF-induced heating, RF power, and B_1+rms_ was shown to vary with RF coils and Tx/Rx combinations: two Tx/Rx volume coils (a body coil and a head coil) and three Rx-only phased-array coils (a 20-channel head and neck, a 32-channel head, and a 64-channel head and neck) in combination with a Tx/Rx body coil at one *B*_0_ field (3T) [37]. The temperature rise, when stratifying the data for individual RF coils and Tx/Rx combinations, had a high linear relation with both RF power and the square of B_1+rms_ at 3T. However, when combining the data for all RF coils and Tx/Rx combinations, the temperature rise had a better linear relation with RF power (R^2^ = 0.9232) than the square of B_1+rms_ (R^2^ = 0.4945) at 3T, indicating the importance of the specifications of RF coils and Tx/Rx combinations.

In this study, only the most widely used Tx/Rx combinations of RF coils (a body coil and a 20-channel head and neck coil) were used at two *B*_0_ fields (1.5T and 3T) to drastically reduce the number of experiments. Each additional RF coil would add 160 experiments for each *B*_0_ field and 320 experiments for both *B*_0_ fields.

Here, the relationship between the scanner-reported RF power, B_1+rms_, and SAR is evaluated as a function of RF pulse sequences, RF coil Tx/Rx combinations, metallic wavelengths, and ionic concentrations. The RF power and SAR varied with RF pulse sequences, RF coil Tx/Rx combinations, metallic wavelengths, and ionic concentrations, whereas B_1+rms_ varied with RF pulse sequences only, but not with Tx/Rx combinations, metallic wavelengths, and ionic concentrations.

The combination of body coil and head coil for transmit and receive had a significantly large effect on RF power, specific absorption rate, transmit reference amplitude, transmit frequency, and absorption ratio due to *B*_1_ shimming, which was available at 3T but not at 1.5T. The RF power and SAR for B_1+rms_ corresponding to a prescribed flip angle change significantly with the RF adjustment strategies and *B*_1_ shimming algorithms [65].

The RF transmit field (*B*_1_) is described by three components: the positive component (*B*_1_*^+^*), the negative component (*B*_1_*^−^*), and the magnitude of both positive and negative components (|*B*_1_|). The effective field that flips the spins in the rotating frame of reference in synchronization with the precession of the spins is the *B*_1_*^+^* field, and it is defined as the time-averaged root-mean-square of the spatially averaged field (B_1+rms_) in the central axial slab [66]. The scanner adjusts the RF power in the transmit coil during the pre-scan mode until the desired *B*_1_*^+^* field is reached for the prescribed flip angle [67,68,69]. As part of RF adjustments, the *B*_1_ shimming (*B*_1_ and *E* field) process adjusts the RF power per RF coil Tx/Rx combinations and RF polarization while maintaining the *B*_1_*^+^* field required for the prescribed flip angle [70,71,72]. This changes the relationship between the RF power and B_1+rms_ depending on the RF coils, Tx/Rx combinations, and experimental conditions [37,38,39,40]. As a result, RF power changes, but *B*_1_ remains nearly the same for the Tx/Rx combinations, metallic wavelengths, and ionic concentrations.

As shown in Table 1 and Table 2, the *dB*_1_/*dθ* did not change, whereas *dP*/*dB*_1_^2^ and *dP*/*dS_w_* changed with the experimental parameters such as Tx/Rx (3T), metallic wavelengths, and ionic concentrations. The slopes of *dP*/*dB*_1_^2^ and *dP*/*dS_w_* varied with conductive metallic wavelengths and ionic concentrations at both 1.5T and 3T, but varied markedly with Tx/Rx combinations at 3T due to *B*_1_ shimming. This can be seen by comparing the *dP*/*dB*_1_^2^ in Figure 2 and Figure 3 and the *dP*/*dS_w_* in Figure 4 and Figure 5 for 1.5T and 3T, respectively.

B_1+rms_ varied linearly with *θ*, proportional to Equation (1), independent of the *B*_0_ field, Tx/Rx, L, and C when using the same RF pulse sequences. Initially, the differences in the average *dB*_1_/*dθ* were observed between the two *B*_0_ fields as 3.5 nT/deg at 1.5T and 9.5 nT/deg at 3T (Table 1 and Table 2). This was further investigated with matched RF sequences (equal *N_S_*, SB, and *T_R_*) and it was found that the difference was due to differences in the prescription of SB and *T_R_* in the nominal RF pulse sequences at 1.5T and 3T (Table 3). Note the effects of SB and *T_R_* of 370 ms at 1.5T vs. no SB and the *T_R_* of 250 ms at 3T, as shown in Table 1, Table 2 and Table 3 at 1.5T and 3T. When SB was on, the slope *dB*_1_/*dθ* decreased, but the intercept *B*_1_(0) increased at both 1.5T and 3T. Without SB, *dB*_1_/*dθ* was equal at both *B*_0_ fields (1.5T and 3T) at equal or scaled *T_R_* (Table 3). The square of the ratios of *dB*_1_/*dθ* scaled with the ratios of 1/*T_R_*; for example, (9.6/7.9)^2^ equals (370/250), as shown in Table 3. *P* varied linearly with *B*_1_^2^ and 1/*T_R_*, proportional to Equations (4) and (5) at both 1.5T and 3T. The *dB*_1_/*dθ* did not change with Tx/Rx combinations, but it changed with *N_S_*, SB, and *T_R_*. Since the B_1+rms_ in RF pulse sequences with SB is not sensitive to the presence or absence of metallic implants, ionic solutions, implant wavelengths, or ionic concentrations, care must be taken to avoid SB because SB increases both *P* and B_1+rms_ significantly.

Since SAR is derived from RF power, their ratio can yield the absorption ratio as *α* = *m*·SAR_wb_/*P* from Equation (3), or *α* = *m*/(*dP*/*dS_wb_*) from Table 1 and Table 2, which can reveal information about SAR computing algorithms of various scanners. A clear distinction must be made between measured and scanner-reported SAR when calculating the *α* because the scanner-reported SAR may yield *α* values greater than unity (*α* > 1, *P_abs_* > *P*) that are not physically possible. The actual SAR and *α* can be calculated by measuring the electric field or the initial temperature rate for incident *P* as in Equations (2) and (3), respectively. The scanner-reported head and whole-body SAR scaled linearly with each other across all experiments, depending on the Tx/Rx combinations. Even though the scanners maintained constant SAR ratios throughout the experiments, a 68 kg patient weight and a small phantom in the head coil might affect the SAR computation. However, using the same phantom size in all experiments shows the variation of parameters with conductive implants and ionic solutions.

As shown in Figure 6 and Figure 7, RF power and SAR changed with flip angles, Tx/Rx (3T), implant wavelength, and ionic concentration, whereas B_1+rms_ changed only with flip angles but not with Tx/Rx, implant wavelength, and ionic concentration. The transmit reference amplitude, transmit frequency, and absorption ratio did not change with flip angle but changed with Tx/Rx (3T), implant wavelength, and ionic concentration. There were more scattered data and outliers observed at 3T than at 1.5T, mainly due to the RF adjustments and *B*_1_ shimming effects present at 3T but not at 1.5T.

While RF power and SAR both vary with the experimental conditions, the ratio between RF power and SAR is highly variable and unpredictable with the experimental conditions. Also, scanner-reported SAR computation for the RF pulse sequences may vary with vendor-dependent algorithms and software versions. These experiments show that the most sensitive parameter appears to be the RF power, as it varies significantly with the experimental conditions for a given B_1+rms_, which varies only with the RF pulse sequence but not the experimental conditions. RF power is most responsive to the presence or absence of conductive metallic implants and ionic solutions, metallic wavelengths, and ionic concentrations. However, RF power also varies with RF coils, RF pulse sequences, RF adjustment strategies (*B*_1_ shimming), as well as patient size and weight. This presents a complex problem for the implementation of RF power in safety standards and technical specifications such as ISO/TS 10974 across MRI systems, implants, and patients [33].

The relationship between RF power, B_1+rms_, and SAR depends on the RF pulse sequences, RF coils, Tx/Rx combinations, RF adjustments, and *B*_1_ shimming strategies, the presence or absence of metallic implants and ionic solutions, implant wavelengths, and ionic concentrations. Experimental measurements are consistent with the simple theoretical formulations through the instrumentation proportionality constants, which can be determined for each of the RF coils and RF pulse sequences experimentally.

The limitation of this study is that two MRI systems (Siemens Aera and Prisma) with the same software versions (Syngo MR XA30A), two RF coil Tx/Rx combinations (BC/BC and BC/20-H/N), a simple metallic conductor (CCA wire), and a simple conductive phantom (H_2_O-NaCl solutions) were used to study the relations between RF power, B_1+rms,_ and SAR. Further tests may be necessary to verify the results across MRI systems, RF coils, implants, and patients.

This experimental method provides a means to simultaneously evaluate the relationship between the scanner-reported RF power, B_1+rms_, and SAR varying with RF pulse sequences, RF coils, Tx/Rx combinations, metallic implant wavelengths, and ionic concentrations during MRI. This method can be used with temperature measurements to evaluate specifically each of the MRI systems, RF coils, RF pulse sequences, conductive metallic implants, and tissue-equivalent conductive ionic solutions to optimize parameters for implant safety.

## 5. Conclusions

The scanner-reported parameters provide noninvasive means and complementary measurements that can be improved and combined to improve RF safety. B_1+rms_ is insensitive to the presence or absence of conductive metallic implants and ionic solutions, metallic wavelengths, ionic concentrations, RF coils, and Tx/Rx combinations. B_1+rms_ alone may not be sufficient to monitor and control RF safety. B_1+rms_ varies with flip angle, independent of the *B*_0_ field, RF coils, Tx/Rx combinations, metallic wavelengths, and ionic concentrations using the same RF pulse sequences. The scanner-reported SAR scales linearly with the scanner-reported average RF power and the square of flip angles, but with different absorption ratios varying with experimental conditions, and remains unpredictable. The scanner-reported average RF power is responsive to the experimental conditions and remains the most sensitive parameter to consider in relation to temperature and improving SAR computation as complementary to B_1+rms_ to monitor and control RF safety. The scanner-reported RF power is sensitive to RF adjustments, metallic wavelengths, and ionic concentrations. Since the RF power was highly associated with RF heating and temperature rise of different magnitudes varying with individual RF coils and B_1_ shimming in a previous study, the response in RF power with experimental conditions may be considered and further studied in addition to B_1+rms_ to improve the development of standards and specifications for implant safety [37,38,39,40]. Because these three scanner-reported parameters, RF power, B_1+rms_, and SAR, are noninvasive and readily available on the scanners, understanding the fundamental relationships between them and the temperature across the MRI systems, implants, and patients can help improve implant safety during MRI.

Prescribing RF saturation bands in RF pulse sequences increases RF power, B_1+rms_, and SAR by orders of magnitude, especially when using low-power RF pulse sequences; therefore, saturation bands must be turned off when imaging patients with conductive metallic implants. The RF power, B_1+rms_, and SAR increase linearly with increasing number of slices and decrease with increasing repetition time.

## Figures and Tables

**Table 1:** Linear regression results (slope and intercept with standard errors) for B_1+rms_ vs. *θ*, *P* vs. the square of B_1+rms_, and *P* vs. SAR_wb_ for RF coils, Tx/Rx, L, and C at 1.5T.

	Aera 1.5T	*B*_1_ vs. *θ*		*P* vs. *B*_1_^2^		*P* vs. SAR_wb_	
No.	RF Coils Tx/Rx (L, C)	*dB*_1_/*dθ* [nT/deg]	*B*_1_ (0) [nT]	*dP*/*dB*_1_^2^ [W/μT^2^]	*P* (0) [W]	*dP*/*dS_wb_* [kg/*α*]	*P* (0) [W]
1	BC/BC (0 cm, 0%)	3.5079 ± 0.2423	839.2774 ± 15.4182	8.9448 ± 0.0038	−0.0072 ± 0.0042	462.2415 ± 0.1941	−0.0072 ± 0.0042
2	BC/20-H/N (0 cm, 0%)	3.5079 ± 0.2423	839.2774 ± 15.4182	8.9448 ± 0.0038	−0.0072 ± 0.0042	462.2415 ± 0.1941	−0.0072 ± 0.0042
3	BC/BC (0 cm, 1%)	3.4941 ± 0.2413	835.9820 ± 15.3576	10.6169 ± 0.0092	0.0071 ± 0.0102	516.9831 ± 0.4459	0.0071 ± 0.0102
4	BC/20-H/N (0 cm, 1%)	3.4941 ± 0.2413	835.9820 ± 15.3576	10.6169 ± 0.0092	0.0071 ± 0.0102	516.9831 ± 0.4459	0.0071 ± 0.0102
5	BC/BC (0 cm, 2%)	3.4951 ± 0.2414	836.2311 ± 15.3622	14.5225 ± 0.0108	−0.0084 ± 0.0120	699.8340 ± 0.5193	−0.0084 ± 0.0120
6	BC/20-H/N (0 cm, 2%)	3.4951 ± 0.2414	836.2311 ± 15.3622	14.5225 ± 0.0108	−0.0084 ± 0.0120	699.8340 ± 0.5193	−0.0084 ± 0.0120
7	BC/BC (0 cm, 3%)	3.4965 ± 0.2415	836.5594 ± 15.3683	22.9795 ± 0.0045	−0.0113 ± 0.0050	849.6723 ± 0.1650	−0.0113 ± 0.0050
8	BC/20-H/N (0 cm, 3%)	3.4965 ± 0.2415	836.5594 ± 15.3683	22.9795 ± 0.0045	−0.0113 ± 0.0050	849.6723 ± 0.1650	−0.0113 ± 0.0050
9	BC/BC (26 cm, 0%)	3.5026 ± 0.2419	838.0267 ± 15.3952	8.4311 ± 0.0071	0.0036 ± 0.0079	433.1065 ± 0.3639	0.0036 ± 0.0079
10	BC/20-H/N (26 cm, 0%)	3.5026 ± 0.2419	838.0267 ± 15.3952	8.4311 ± 0.0071	0.0036 ± 0.0079	433.1065 ± 0.3639	0.0036 ± 0.0079
11	BC/BC (26 cm, 1%)	3.4981 ± 0.2416	836.9313 ± 15.3751	11.0085 ± 0.0074	0.0071 ± 0.0083	531.5672 ± 0.3584	0.0071 ± 0.0083
12	BC/20-H/N (26 cm, 1%)	3.4981 ± 0.2416	836.9313 ± 15.3751	11.0085 ± 0.0074	0.0071 ± 0.0083	531.5672 ± 0.3584	0.0071 ± 0.0083
13	BC/BC (26 cm, 2%)	3.4954 ± 0.2414	836.3007 ± 15.3635	15.9331 ± 0.0000	−0.0123 ± 0.0000	761.7769 ± 0.0000	−0.0123 ± 0.0000
14	BC/20-H/N (26 cm, 2%)	3.4954 ± 0.2414	836.3007 ± 15.3635	15.9331 ± 0.0000	−0.0123 ± 0.0000	761.7769 ± 0.0000	−0.0123 ± 0.0000
15	BC/BC (26 cm, 3%)	3.4999 ± 0.2417	837.3756 ± 15.3833	21.0570 ± 0.0055	−0.0050 ± 0.0062	911.6638 ± 0.2394	−0.0050 ± 0.0062
16	BC/20-H/N (26 cm, 3%)	3.4999 ± 0.2417	837.3756 ± 15.3833	21.0570 ± 0.0055	−0.0050 ± 0.0062	911.6638 ± 0.2394	−0.0050 ± 0.0062
17	BC/BC (52 cm, 0%)	3.4970 ± 0.2415	836.6827 ± 15.3706	20.5929 ± 0.0084	0.0003 ± 0.0094	933.1221 ± 0.3820	0.0003 ± 0.0094
18	BC/20-H/N (52 cm, 0%)	3.4970 ± 0.2415	836.6827 ± 15.3706	20.5929 ± 0.0084	0.0003 ± 0.0094	933.1221 ± 0.3820	0.0003 ± 0.0094
19	BC/BC (52 cm, 1%)	3.4971 ± 0.2415	836.7101 ± 15.3710	13.7038 ± 0.0113	−0.0074 ± 0.0125	676.6085 ± 0.5554	−0.0074 ± 0.0125
20	BC/20-H/N (52 cm, 1%)	3.4971 ± 0.2415	836.7101 ± 15.3710	13.7038 ± 0.0113	−0.0074 ± 0.0125	676.6085 ± 0.5554	−0.0074 ± 0.0125
21	BC/BC (52 cm, 2%)	3.4966 ± 0.2415	836.5728 ± 15.3684	18.0586 ± 0.0108	−0.0028 ± 0.0120	881.7400 ± 0.5257	−0.0028 ± 0.0120
22	BC/20-H/N (52 cm, 2%)	3.4966 ± 0.2415	836.5728 ± 15.3684	18.0586 ± 0.0108	−0.0028 ± 0.0120	881.7400 ± 0.5257	−0.0028 ± 0.0120
23	BC/BC (52 cm, 3%)	3.4944 ± 0.2413	836.0545 ± 15.3590	21.4671 ± 0.0085	−0.0165 ± 0.0094	896.9432 ± 0.3538	−0.0165 ± 0.0094
24	BC/20-H/N (52 cm, 3%)	3.4944 ± 0.2413	836.0545 ± 15.3590	21.4671 ± 0.0085	−0.0165 ± 0.0094	896.9432 ± 0.3538	−0.0165 ± 0.0094
25	BC/BC (104 cm, 0%)	3.5023 ± 0.2419	837.9548 ± 15.3939	12.8766 ± 0.0055	0.0109 ± 0.0062	663.8650 ± 0.2847	0.0109 ± 0.0062
26	BC/20-H/N (104 cm, 0%)	3.5023 ± 0.2419	837.9548 ± 15.3939	12.8766 ± 0.0055	0.0109 ± 0.0062	663.8650 ± 0.2847	0.0109 ± 0.0062
27	BC/BC (104 cm, 1%)	3.4937 ± 0.2413	835.8888 ± 15.3560	14.5720 ± 0.0061	0.0022 ± 0.0068	723.3868 ± 0.3034	0.0022 ± 0.0068
28	BC/20-H/N (104 cm, 1%)	3.4937 ± 0.2413	835.8888 ± 15.3560	14.5720 ± 0.0061	0.0022 ± 0.0068	723.3868 ± 0.3034	0.0022 ± 0.0068
29	BC/BC (104 cm, 2%)	3.4948 ± 0.2414	836.1600 ± 15.3610	21.8251 ± 0.0118	0.0040 ± 0.0131	882.8685 ± 0.4766	0.0040 ± 0.0131
30	BC/20-H/N (104 cm, 2%)	3.4948 ± 0.2414	836.1600 ± 15.3610	21.8251 ± 0.0118	0.0040 ± 0.0131	882.8685 ± 0.4766	0.0040 ± 0.0131
31	BC/BC (104 cm, 3%)	3.4958 ± 0.2414	836.3860 ± 15.3650	23.3877 ± 0.0111	−0.0118 ± 0.0123	841.0524 ± 0.3987	−0.0117 ± 0.0123
32	BC/20-H/N (104 cm, 3%)	3.4958 ± 0.2414	836.3860 ± 15.3650	23.3877 ± 0.0111	−0.0118 ± 0.0123	841.0524 ± 0.3987	−0.0117 ± 0.0123

**Table 2:** Linear regression results (slope and intercept with standard errors) for B_1+rms_ vs. *θ*, *P* vs. the square of B_1+rms_, and *P* vs. SAR_wb_ for RF coils, Tx/Rx, L, and C at 3T.

	Prisma 3T	*B*_1_ vs. *θ*		*P* vs. *B*_1_^2^		*P* vs. SAR_wb_	
No.	RF Coils Tx/Rx (L, C)	*dB*_1_/*dθ* [nT/deg]	*B*_1_ (0) [nT]	*dP*/*dB*_1_^2^ [W/μT^2^]	*P* (0) [W]	*dP*/*dS_wb_* [kg/*α*]	*P* (0) [W]
1	BC/BC (0 cm, 0%)	9.4412 ± 0.0000	0.0000 ± 0.0000	3.2715 ± 0.0026	−0.0010 ± 0.0011	76.6099 ± 0.0610	−0.0010 ± 0.0011
2	BC/20-H/N (0 cm, 0%)	9.4173 ± 0.0000	0.0000 ± 0.0000	3.4138 ± 0.0029	−0.0021 ± 0.0012	79.9424 ± 0.0686	−0.0021 ± 0.0012
3	BC/BC (0 cm, 1%)	9.4790 ± 0.0000	0.0000 ± 0.0000	5.5152 ± 0.0038	0.0050 ± 0.0016	110.5703 ± 0.0759	0.0050 ± 0.0016
4	BC/20-H/N (0 cm, 1%)	9.4187 ± 0.0000	0.0000 ± 0.0000	7.8605 ± 0.0049	−0.0001 ± 0.0021	109.3546 ± 0.0685	−0.0001 ± 0.0021
5	BC/BC (0 cm, 2%)	9.5024 ± 0.0000	0.0000 ± 0.0000	7.5683 ± 0.0058	0.0043 ± 0.0025	110.5145 ± 0.0840	0.0043 ± 0.0025
6	BC/20-H/N (0 cm, 2%)	9.4298 ± 0.0000	0.0000 ± 0.0000	15.9123 ± 0.0079	−0.0006 ± 0.0034	88.5539 ± 0.0441	−0.0006 ± 0.0034
7	BC/BC (0 cm, 3%)	9.5023 ± 0.0000	0.0000 ± 0.0000	7.7695 ± 0.0070	0.0008 ± 0.0030	111.1972 ± 0.1007	0.0008 ± 0.0030
8	BC/20-H/N (0 cm, 3%)	9.4345 ± 0.0000	0.0000 ± 0.0000	19.7675 ± 0.0045	−0.0040 ± 0.0019	75.7073 ± 0.0171	−0.0040 ± 0.0019
9	BC/BC (13 cm, 0%)	9.4090 ± 0.0000	0.0000 ± 0.0000	3.0122 ± 0.0000	0.0000 ± 0.0000	70.5385 ± 0.0000	0.0000 ± 0.0000
10	BC/20-H/N (13 cm, 0%)	9.4211 ± 0.0000	0.0000 ± 0.0000	3.0986 ± 0.0029	0.0021 ± 0.0012	72.5624 ± 0.0685	0.0021 ± 0.0012
11	BC/BC (13 cm, 1%)	9.4699 ± 0.0000	0.0000 ± 0.0000	5.6690 ± 0.0026	0.0010 ± 0.0011	110.5300 ± 0.0505	0.0010 ± 0.0011
12	BC/20-H/N (13 cm, 1%)	9.4216 ± 0.0000	0.0000 ± 0.0000	9.3126 ± 0.0055	−0.0039 ± 0.0024	109.1341 ± 0.0649	−0.0039 ± 0.0024
13	BC/BC (13 cm, 2%)	9.5155 ± 0.0000	0.0000 ± 0.0000	7.1181 ± 0.0061	−0.0023 ± 0.0026	110.8938 ± 0.0943	−0.0023 ± 0.0026
14	BC/20-H/N (13 cm, 2%)	9.4465 ± 0.0000	0.0000 ± 0.0000	17.7112 ± 0.0026	−0.0010 ± 0.0011	81.2992 ± 0.0119	−0.0010 ± 0.0011
15	BC/BC (13 cm, 3%)	9.4835 ± 0.0000	0.0000 ± 0.0000	8.6972 ± 0.0055	−0.0039 ± 0.0024	111.0424 ± 0.0697	−0.0039 ± 0.0024
16	BC/20-H/N (13 cm, 3%)	9.4424 ± 0.0000	0.0000 ± 0.0000	27.0554 ± 0.0076	0.0005 ± 0.0033	69.3702 ± 0.0195	0.0005 ± 0.0033
17	BC/BC (26 cm, 0%)	9.4294 ± 0.0000	0.0000 ± 0.0000	1.5187 ± 0.0056	−0.0029 ± 0.0024	35.5636 ± 0.1306	−0.0029 ± 0.0024
18	BC/20-H/N (26 cm, 0%)	9.3764 ± 0.0000	0.0000 ± 0.0000	1.2947 ± 0.0026	−0.0010 ± 0.0011	30.3195 ± 0.0618	−0.0010 ± 0.0011
19	BC/BC (26 cm, 1%)	9.4882 ± 0.0000	0.0000 ± 0.0000	6.0481 ± 0.0030	−0.0011 ± 0.0013	111.6514 ± 0.0562	−0.0011 ± 0.0013
20	BC/20-H/N (26 cm, 1%)	9.4626 ± 0.0000	0.0000 ± 0.0000	12.0841 ± 0.0094	−0.0002 ± 0.0040	107.6392 ± 0.0836	−0.0002 ± 0.0040
21	BC/BC (26 cm, 2%)	9.5022 ± 0.0000	0.0000 ± 0.0000	6.8777 ± 0.0061	0.0030 ± 0.0027	110.9341 ± 0.0989	0.0030 ± 0.0027
22	BC/20-H/N (26 cm, 2%)	9.4645 ± 0.0000	0.0000 ± 0.0000	24.4226 ± 0.0051	0.0018 ± 0.0022	71.0778 ± 0.0148	0.0018 ± 0.0022
23	BC/BC (26 cm, 3%)	9.5049 ± 0.0000	0.0000 ± 0.0000	6.4879 ± 0.0029	0.0021 ± 0.0012	111.2169 ± 0.0493	0.0021 ± 0.0012
24	BC/20-H/N (26 cm, 3%)	8.5705 ± 0.5031	39.2144 ± 32.0185	37.1368 ± 0.0061	−0.0011 ± 0.0024	65.2964 ± 0.0106	−0.0011 ± 0.0024
25	BC/BC (52 cm, 0%)	9.4751 ± 0.0000	0.0000 ± 0.0000	3.6826 ± 0.0026	0.0010 ± 0.0011	86.2377 ± 0.0605	0.0010 ± 0.0011
26	BC/20-H/N (52 cm, 0%)	9.4154 ± 0.0000	0.0000 ± 0.0000	4.1841 ± 0.0095	−0.0002 ± 0.0040	97.9804 ± 0.2221	−0.0002 ± 0.0040
27	BC/BC (52 cm, 1%)	9.4833 ± 0.0000	0.0000 ± 0.0000	5.9493 ± 0.0055	−0.0029 ± 0.0024	112.2265 ± 0.1040	−0.0029 ± 0.0024
28	BC/20-H/N (52 cm, 1%)	9.4394 ± 0.0000	0.0000 ± 0.0000	10.4000 ± 0.0038	−0.0050 ± 0.0016	108.2694 ± 0.0398	−0.0050 ± 0.0016
29	BC/BC (52 cm, 2%)	9.4984 ± 0.0000	0.0000 ± 0.0000	7.6349 ± 0.0061	0.0023 ± 0.0026	111.3332 ± 0.0886	0.0023 ± 0.0026
30	BC/20-H/N (52 cm, 2%)	9.4276 ± 0.0000	0.0000 ± 0.0000	19.6711 ± 0.0039	−0.0028 ± 0.0017	75.7476 ± 0.0150	−0.0028 ± 0.0017
31	BC/BC (52 cm, 3%)	9.4731 ± 0.0000	0.0000 ± 0.0000	9.3410 ± 0.0082	−0.0010 ± 0.0035	116.2077 ± 0.1020	−0.0010 ± 0.0035
32	BC/20-H/N (52 cm, 3%)	9.4726 ± 0.0000	0.0000 ± 0.0000	23.9288 ± 0.0049	0.0001 ± 0.0021	71.2071 ± 0.0145	0.0001 ± 0.0021

**Table 3:** Linear regression results (slope and intercept with R^2^) for B_1+rms_ vs. *θ* and *P* vs. the square of B_1+rms_ as a function of *N_S_*, SB, Tx/Rx, and *T_R_* for a standard phantom with an aqueous solution of 5 g/L NaCl and 3.75 g/L NiSO_4_·6H_2_O without an implant at 1.5T and 3T.

				***B*_0_ = 1.5T**					
				***B*_1_ vs. *θ***			***P* vs. *B*_1_^2^**		
***N_S_***	**SB**	**Tx/Rx**	***T_R_*** **[ms]**	***dB*_1_/*dθ*** **[nT/deg]**	***B*_1_ (0)** **[nT]**	**R^2^**	***dP*/*dB*_1_^2^** **[W/μT^2^]**	***P* (0)** **[W]**	**R^2^**
1	1	2	250	2.4824	593.9244	0.9859	9.2459	0.0017	1.0000
1	1	2	370	2.0405	488.2029	0.9859	9.3522	−0.0090	1.0000
3	1	2	250	4.2996	1028.7073	0.9859	9.2561	−0.0141	1.0000
3	1	2	370	3.5343	845.5922	0.9859	9.3251	0.0056	1.0000
1	2	2	250	5.5634	0.0000	1.0000	9.2459	0.0010	1.0000
1	2	2	370	4.5731	0.0000	1.0000	9.3002	0.0043	1.0000
3	2	2	250	9.6361	0.0000	1.0000	9.2561	−0.0028	1.0000
3	2	2	370	7.9208	0.0000	1.0000	9.3154	0.0050	1.0000
3	1	1	370	3.5332	845.3501	0.9859	9.3207	0.0085	1.0000
3	2	1	370	7.9185	0.0000	1.0000	9.3305	0.0006	1.0000
				***B*_0_ = 3T**					
				***B*_1_ vs. *θ***			***P* vs. *B*_1_^2^**		
***N_S_***	**SB**	**Tx/Rx**	***T_R_*** **[ms]**	***dB*_1_/*dθ*** **[nT/deg]**	***B*_1_ (0)** **[nT]**	**R^2^**	***dP*/*dB*_1_^2^** **[W/μT^2^]**	***P* (0)** **[W]**	**R^2^**
1	1	2	250	2.8633	462.4106	0.9881	7.7708	0.0034	1.0000
1	1	2	370	2.3537	380.0992	0.9881	7.8077	0.0064	1.0000
3	1	2	250	4.9595	800.9186	0.9881	9.1130	−1.4165	0.9867
3	1	2	370	4.0766	658.3511	0.9881	7.8374	−0.0033	1.0000
1	2	2	250	5.5254	0.0000	1.0000	7.8220	−0.0032	1.0000
1	2	2	370	4.5418	0.0000	1.0000	7.8143	0.0032	1.0000
3	2	2	250	9.5703	0.0000	1.0000	7.7945	−0.0033	1.0000
3	2	2	370	7.8667	0.0000	1.0000	7.8374	0.0017	1.0000
3	1	1	250	4.9723	802.9972	0.9881	7.9491	0.0007	1.0000
3	2	1	250	9.5951	0.0000	1.0000	7.9543	−0.0019	1.0000

SB (1: on, 2: off), Tx/Rx (1: BC/BC, 2: BC/20-H/N).

## Data Availability

The original contributions presented in this study are included in the article. Further inquiries can be directed to the corresponding author.

## Abbreviations

The following abbreviations are used in this manuscript:

MRI	Magnetic Resonance Imaging
RF	Radiofrequency
*E*	Root-mean-square electric field, Volts/m, or V/m
B_1+rms_	Root-mean-square magnetic field, micro-Tesla, nano-Tesla or μT, nT
*B*_0_	Magnetic field strength, Tesla or T
*γ*	Gyromagnetic ratio, rads/s·T
*P*	RF power, Watts or kilowatts, W or kW
Tx/Rx	Transmit and receive
BC	Body Coil
20-H/N	20 channel Head and Neck coil
*m*	Patient weight, kg
SAR	Specific absorption rate, W/kg
SAR_wb_	Whole body SAR, W/kg
SAR_h_	Head SAR, W/kg
H_2_O-NaCl	Water and sodium chloride
*σ*	Electrical conductivity, Siemens per meter or S/m
*θ* or FA	Flip angle, rads or degrees
*λ*	Wavelength, cm
L	Length, cm
C	Concentration, mM or %
*α*	Calculated absorption ratio, dimensionless
*D*	Duty cycle, dimensionless
*ω*	Angular frequency, rads/s
CIED	Cardiac implantable electronic devices
DBS	Deep brain stimulation
*t_p_*	Pulse length, milliseconds or ms
*T_R_*	Repetition time, milliseconds or ms
*N_S_*	Number of slices, dimensionless
SB	Saturation bands, millimeters or mm
TxRA	Transmit reference amplitude, Volts or V
TxF	Transmit frequency, Hertz or Mega Hertz, Hz or MHz
